# Supplementary material for: A Humanized Yeast Phenomic Model of Deoxycytidine Kinase to Predict Genetic Buffering of Nucleoside Analog Cytotoxicity
Source: Genes (Basel). 2019 Sep 30;10(10):770. doi: 10.3390/genes10100770 (PMC6826991; doi:10.3390/genes10100770)
Supplement: Supplementary file 1 [file genes-10-00770-s001.zip › Additional_Files/Additional_File7_GOTermHeatmaps.zip!/Additional_File7_GOTermHeatmaps/telomere_capping.pdf]

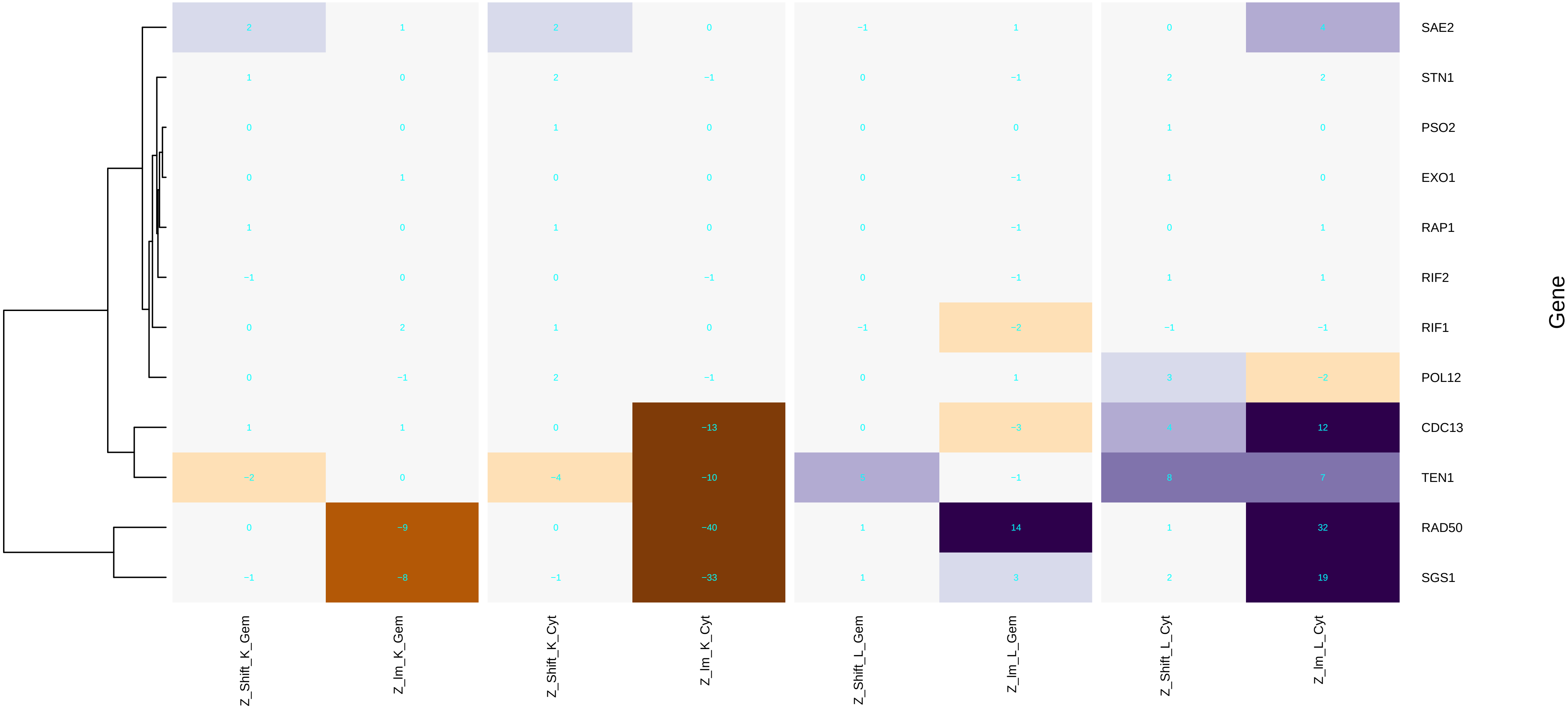
Gene
Z_Shift_K_Gem
Z_Im_K_Gem
Z_Shift_K_Cyt
Z_Im_K_Cyt
Z_Shift_L_Gem
Z_Im_L_Gem
Z_Shift_L_Cyt
Z_Im_L_Cyt
SAE2 2 1 2 0 −1 1 0 4
STN1 1 0 2 −1 0 −1 2 2
PSO2 0 0 1 0 0 0 1 0
EXO1 0 1 0 0 0 −1 1 0
RAP1 1 0 1 0 0 −1 0 1
RIF2 −1 0 0 −1 0 −1 1 1
RIF1 0 2 1 0 −1 −2 −1 −1
POL12 0 −1 2 −1 0 1 3 −2
CDC13 1 1 0 −13 0 −3 4 12
TEN1 −2 0 −4 −10 5 −1 8 7
RAD50 0 −9 0 −40 1 14 1 32
SGS1 −1 −8 −1 −33 1 3 2 19
